# Melatonin mitigates type 1 diabetes‐aggravated cerebral ischemia‐reperfusion injury through anti‐inflammatory and anti‐apoptotic effects

**DOI:** 10.1002/brb3.3118

**Published:** 2023-06-16

**Authors:** Qian Xu, Raymond Tak Fai Cheung

**Affiliations:** ^1^ Department of Medicine, School of Clinical Medicine, Li Ka Shing Faculty of Medicine The University of Hong Kong Hong Kong; ^2^ Research Centre of Heart, Brain, Hormone & Healthy Aging, Li Ka Shing Faculty of Medicine The University of Hong Kong Hong Kong

**Keywords:** anti‐apoptosis, anti‐inflammatory, diabetes mellitus, ischemia‐reperfusion, melatonin, neuroprotective

## Abstract

**Introduction:**

Cerebral ischemia and diabetes mellitus (DM) are common diseases that often coexist and interact with each other. DM doubles the risk of ischemic stroke, and cerebral ischemia causes stress‐induced hyperglycemia. Most experimental stroke studies used healthy animals. Melatonin is neuroprotective against cerebral ischemia–reperfusion injury (CIRI) in non‐DM, normoglycemic animals through anti‐oxidant effect, anti‐inflammation, and anti‐apoptosis. Previous studies have also reported a negative correlation between hyperglycemia and urinary melatonin metabolite.

**Objectives:**

The present study investigated the effects of type 1 DM (T1DM) on CIRI in rats and the role of melatonin against CIRI in T1DM animals.

**Results:**

Our results revealed that T1DM aggravated CIRI, leading to greater weight loss, increased infarct volume, and worse neurological deficit. T1DM aggravated the post‐CIRI activation of nuclear factor kappa B (NF‐κB) pathway and increase in pro‐apoptotic markers. A single intraperitoneal injection of melatonin at 10 mg/kg given 30 min before ischemia onset attenuated CIRI in T1DM rats, resulting in less weight loss, decreased infarct volume, and milder neurological deficit when compared with the vehicle group. Melatonin treatment achieved anti‐inflammatory and anti‐apoptotic effects with reduced NF‐κB pathway activation, reduced mitochondrial cytochrome C release, decreased calpain‐mediated spectrin breakdown product (SBDP), and decreased caspase‐3‐mediated SBDP. The treatment also led to fewer iNOS+ cells, milder CD‐68+ macrophage/microglia infiltration, decreased TUNEL+ apoptotic cells, and better neuronal survival.

**Conclusions:**

T1DM aggravates CIRI. Melatonin treatment is neuroprotective against CIRI in T1DM rats via anti‐inflammatory and anti‐apoptotic effects.

## Graphical abstract

The present study investigated the effects of type 1 DM (T1DM) on cerebral ischemia–reperfusion injury (CIRI) in rats and the role of melatonin against CIRI in T1DM animals. T1DM aggravates CIRI, and melatonin treatment is neuroprotective against CIRI in T1DM rats via anti‐inflammatory and anti‐apoptotic effects.

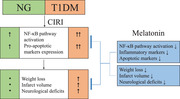

## 1 INTRODUCTION

Stroke is the second leading cause of mortality globally and a leading cause of long‐term disability in high‐income countries (Luitse et al., 2012), and ischemic stroke accounts for 65% of all strokes (Krishnamurthi et al., 2020). Rapid reversal of cerebral ischemia through reperfusion via intravenous thrombolysis and/or endovascular thrombectomy is highly effective in salvaging ischemic tissue and reducing disability if initiated within a short time window (Campbell et al., 2019). At the same time, reperfusion following ischemia provokes reperfusion injury via excitotoxicity, oxidative stress, mitochondrial dysfunction, inflammation, and apoptosis (Hotchkiss et al., 2009; Lo et al., 2003). Calcium ion dysregulation contributes to cell death via activation of calcium‐dependent proteases such as calpain (Bartus et al., 1998; Cheng et al., 2018; Inserte et al., 2012). Activated caspase‐3 is at the convergence point for different signaling pathways in apoptosis (Broughton et al., 2009). Most experimental studies on cerebral ischemia–reperfusion injury (CIRI) use healthy animals without vascular risk factors (Dirnagl et al., 1999; Fluri et al., 2015). Nevertheless, most strokes occur in people with one or more risk factors such as hypertension, diabetes mellitus (DM), ischemic heart disease, hypercholesterolemia, smoking, and old age (Campbell et al., 2019).

According to the International Diabetes Federation, globally around 8.8% of adults have DM (International Diabetes Federation, 2015). Both DM and ischemic stroke are common, and they often coexist and interact with each other in the same individuals. Hyperglycemia occurs in 30%−40% of ischemic stroke patients including some without a prior history of DM (Luitse et al., 2012). Hyperglycemia upon admission is associated with a worse functional outcome, a larger infarct size, and a higher mortality rate (Kruyt et al., 2010), and these associations are independent of other predictors of poor prognosis such as age, diabetic status, and stroke severity (Kruyt et al., 2010). The inferior outcomes may result from delayed recanalization and increased reperfusion injury due to oxidative stress and inflammation (Luitse et al., 2012). The relationship between glucose metabolism and ischemic stroke is bidirectional (Luitse et al., 2012). DM doubles the risk of ischemic stroke despite correction for other risk factors, and acute stroke per se is sufficient in producing stress‐induced hyperglycemia (Luitse et al., 2012).

Advanced glycation end products (AGEs) formed within hyperglycemic environments play a key role in the pathophysiology of DM‐related vascular disease (Goldin et al., 2006). Activation of receptors for AGEs (RAGE) upregulates nuclear factor kappa light chain enhancer of activated B cells (NF‐κB), which is an important transcription factor involved in cellular responses to various stimuli, and subsequently NF‐κB target genes (Goldin et al., 2006), leading to differential effects on nitric oxide synthase (NOS) activities. The latter include inhibition of endothelial NOS and activation of inducible NOS (iNOS), resulting in endothelial dysfunction and generation of reactive oxygen species (ROS), respectively (Goldin et al., 2006). NF‐κB inhibitor alpha (IκBα) is a key cellular inhibitory protein of this transcription factor. Thus, hyperglycemia disturbs the balance between ROS and antioxidants and constitutes an oxidative stress (Luitse et al., 2012). Mitochondria play a key role in ROS generation and apoptosis in stroke (Broughton et al., 2009). Mitochondrial ROS generation induces the dissociation of cytochrome C from the mitochondrial inner membrane, and the release of cytochrome C and other apoptotic proteins from the mitochondria into cytosol to initiate the intrinsic pathway of apoptosis (Broughton et al., 2009).

The incidence of type 1 DM (T1DM) has been rising over the past 25 years; T1DM can occur at any age, with up to 50% of diagnosis made in adulthood (Dimeglio et al., 2018; Norris et al., 2020). The hyperglycemic environment in T1DM is caused by insulin deficiency from pancreas β‐cell destruction, and T1DM can be induced by a single injection of streptozotocin (STZ) in experimental animals (American Diabetes Association, 2014). The hyperglycemia in type 2 DM is caused by insulin resistance from inability of insulin‐sensitive tissues to respond appropriately to insulin (Galicia‐Garcia et al., 2020).

Melatonin (*N*‐acetyl‐5‐methoxytryptamine) is a neurohormone released from the pineal gland into the cerebrospinal fluid and blood especially at night (Tamtaji et al., 2019). There is recent evidence for its universal production in the mitochondria of all cells (Tan et al., 2016; Venegas et al., 2012) and its general functions as an antioxidant (Reiter et al., 2016; Tan et al., 2016). A negative correlation between hyperglycemia and urinary excretion of melatonin metabolite, 6‐sulfatoxymelatonin, has been reported in both diabetic patients and STZ‐induced T1DM rats (Amaral et al., 2014). Pineal melatonin content decreases sharply after T1DM induction and remains low during the entire experimental period of at least 45 days (Amaral et al., 2014). Melatonin is neuroprotective against CIRI in experimental stroke models in different mammalian species via anti‐oxidative effect, anti‐inflammation, and anti‐apoptosis (Hardeland, 2018; Reiter et al., 2016; Tamtaji et al., 2019). Immediate melatonin treatment in normoglycemic (NG) animals reduces brain infarct volume and neurological deficit, increases neuronal survival, and preserves brain circuit connectivity (Cervantes et al., 2008). Melatonin exerts its neuroprotective effects via direct and indirect antioxidant activity, prevention and reversal of mitochondrial malfunction, and reduction in inflammation, cytoskeletal disorganization, and pro‐apoptotic cell signals (Cervantes et al., 2008). It is unknown if the protective effects of melatonin against CIRI may be affected by hyperglycemia in animals with DM. In the present study, we first examined the detrimental effects of hyperglycemia on CIRI and the underlying mechanisms in T1DM rats. Next, we investigated the protective effects of melatonin treatment against CIRI in T1DM rats.

## 2 METHODS

### 2.1 Animals

Adult male Sprague–Dawley (SD) rats weighing 190−210 g were obtained from the Centre for Comparative Medicine Research (CCMR), the University of Hong Kong, Hong Kong. The experimental protocol (#4470‐17) was reviewed and approved by the Committee on the Use of Live Animals in Teaching and Research of the University of Hong Kong. Rats were housed in a managed light–dark rhythm (a 12:12‐h light–dark cycle, light on at 7:00 a.m.) with controlled temperature (22−24°C) for at least 3 days before commencement of experimental procedures. Altogether 79 rats were used in this study. Rats have free access to food and water. Experimental procedures were performed in accordance with CCMR regulations for the care and use of laboratory animals.

### 2.2 T1DM induction

T1DM was induced in SD rats by a single intraperitoneal injection (IP) of STZ (65 mg/kg; Sigma–Aldrich, St. Louis, MO, USA) freshly prepared in citrate buffer (50 mM, sodium citrate, pH 4.5) as the vehicle. Control NG rats were injected with the vehicle only. Rats were randomly assigned to injection with STZ or citrate buffer. One week later, random glucose concentration of the tail vein blood specimen was checked using a glucometer (Accu‐Chek, Roche Group, Basel, Switzerland). Rats with random blood glucose levels higher than 16.7 mmol/L were considered diabetic (Yan et al., 2012, 2018). Rats not reaching this level of hyperglycemia after STZ injection were excluded from further procedures. Two weeks later, rats underwent middle cerebral artery occlusion (MCAO). Body weights were measured before MCAO and at 24 h after reperfusion.

### 2.3 Right‐sided endovascular MCAO

CIRI was induced by right‐sided endovascular MCAO for 75 or 90 min followed by reperfusion for 24 h. Rats were anesthetized with an IP injection of a mixture of ketamine (67 mg/kg; Alfasan International B.V., Woerden, Netherlands) plus xylazine (6.7 mg/kg; Alfasan International B.V.) before exposure of the right carotid arteries. The right external carotid artery (ECA) was isolated distally after division of its branches using bipolar electric coagulation (GN60; Aesculap AG & Co, Hesse, Germany). Micro clips were temporarily applied to both the common carotid artery and internal carotid artery (ICA). A piece of 4‐*o* monofilament nylon suture (4037PK5Re; Doccol Corporation, Sharon, MA, USA) was inserted via the ECA stump into the right ICA lumen at about 18−20 mm from the carotid bifurcation to occlude the right middle cerebral artery (MCA) at its origin. After 75 or 90 min of focal ischemia, the nylon suture was withdrawn carefully to achieve reperfusion. Rats of the sham MCAO groups underwent the same procedures except for the occlusion of the MCA. After reperfusion, the neck incision was closed with silk sutures, and the rats were allowed to recover from the anesthesia. Buprenorphine (50 μg/kg every 12 h; Reckitt Benckiser Healthcare Ltd, Hull, UK) was given subcutaneously for postoperative pain relief. Enrofloxacin (10 mg/kg; Bayer Ltd, Kiel, Germany) was given intramuscularly for prevention of wound infection. Rectal temperature was kept at 37.0 ± 0.5°C by using a rectal thermostat probe and a thermostatically regulated heating pad. The heart rate and respiratory rate were monitored every 20−30 min during MCAO. At 24 h after reperfusion, the rats were anesthetized with an IP injection of pentobarbital (100 mg/kg; Alfasan International B.V.) before sacrifice to obtain the brain. Rats were excluded from the study if failed MCAO induction or dead before sacrifice.

### 2.4 Monitoring of the regional cerebral blood flow

While under anesthesia for the endovascular MCAO, a burr hole was made on the skull at 2 mm posterior and 5 mm lateral to the bregma on the right side for monitoring the regional cerebral blood flow (CBF) using a laser Doppler flowmeter (MBF3D; Moor Instruments Limited, Axminster, Devon, UK). Successful induction of MCAO was reflected by a drastic decline in the regional CBF to less than 35% of the baseline values. A surge in regional CBF to more than 80% of baseline value upon withdrawal of the nylon suture would reveal reperfusion. Regional CBF data at onset, 30 and 60 min of ischemia, and upon reperfusion were expressed as percentages of the baseline values for analysis.

### 2.5 Drug administration

Some groups of rats received a single IP dose of melatonin (10 mg/kg) or its vehicle 30 min before MCAO onset. Melatonin (Sigma–Aldrich) was first dissolved in dimethyl sulfoxide (DMSO; Sigma–Aldrich) before dilution using normal saline with a final concentration of DMSO <5%. Random glucose concentration of the tail vein blood specimen was checked at 24 h of reperfusion before sacrifice. T1DM rats were randomly assigned to injection with melatonin or 5% DMSO as the vehicle.

### 2.6 Modified neurological severity score

Neurobehavioral performance in motor and sensory functions, reflexes, and balance was assessed using the modified neurological severity score (mNSS) (Lee et al., 2014; Schaar et al., 2010). One point is given for the incomplete achievement of a test item. Higher points indicate more severe neurological deficit. The maximum score is 18 points, and healthy rats score zero points on mNSS.

### 2.7 Rotarod test

Three days before the MCAO, some rats received daily training on a rotating treadmill at 10 revolutions per minute (Barone et al., 1992; Söderpalm et al., 1989). Rats incapable of maintaining a minimum duration of 5 min on the rotating treadmill during training were excluded. The latency to fall off was taken as 5 min at baseline. The test was repeated at 24 h of reperfusion.

### 2.8 Edema‐adjusted brain infarct

The brain between +4 and −8 mm bregma level was cut into 2‐mm thick coronal slices. Slices were reacted with 1.5% triphenyl tetrazolium chloride (Sigma–Aldrich) solution to reveal the infarct. Digital photographs of the coronal slices were obtained by a scanner for infarct size measurement using the Image J system (Image J, v1.51m9; National Institutes of Health, USA). The edema‐adjusted (EA) infarct volume was derived from the integrated infarct volume multiplied by the ratio between contralateral and ipsilateral hemispheric volumes (Nouraee et al., 2019).

### 2.9 Western blot

Cortical regions of the brain between +2 and −2 mm bregma level were collected for homogenization. Protein content was separated by 10%−15% sodium dodecyl sulphate–polyacrylamide gel electrophoresis and then transferred to polyvinylidene fluoride (PVDF) membranes (Bio‐Rad, Hercules, USA). The PVDF membranes were blocked with 10% nonfat milk (Bio‐Rad) for 1 h at room temperature and then incubated with primary antibodies in 5% nonfat milk overnight at 4°C. The membranes were then incubated with corresponding secondary antibodies for 2 h at room temperature for detection of phosphorylated IκBα (p‐IκBα), total‐IκBα, cytochrome C, spectrin breakdown product (SBDP), and β‐actin.

Activation of NF‐κB is indicated by the ratio of p‐IκBα to total IκBα (Viatour et al., 2005). Activations of calpain and caspase‐3 are revealed by specific proteolysis of α‐II‐spectrin (α‐fodrin) into 145‐kDa calpain‐mediated SBDP and 120‐kDa caspase‐3‐specific SBDP, respectively (Wang, 2000; Zhang & Bhavnani, 2006). The blots were imaged with Enhanced Chemiluminescent reagents (GE Healthcare Life Sciences, USA) and recorded by the Imagine System (Bio‐Rad). Protein expressions were analyzed using the Image J system (v1.51m9). The band intensity was adjusted according to the β‐actin band and expressed as a percentage of the sham or vehicle group for analysis.

Primary antibodies against p‐IκBα (1:1000, 9246) and total‐IκBα (1:1000, 4814) were purchased from Cell Signaling Technology (Massachusetts, USA). Primary antibodies against β‐actin (1:1000, sc‐81178) and cytochrome C (1:500, sc‐13561) were purchased from Santa Cruz Biotechnology (Texas, USA). Primary antibody against α‐fodrin (1:4000, BML‐FG6090) was purchased from Enzo Life Science (New York, USA). Horseradish peroxidase‐conjugated rabbit anti‐mouse secondary antibody (1:2000, Nr.P0260) was purchased from Dako (Glostrup, Denmark). Horseradish peroxidase‐conjugated goat anti‐rabbit secondary antibody (1:2000, sc‐2357) was purchased from Santa Cruz Biotechnology.

### 2.10 Immunofluorescence staining

Anesthetized rats were transcardially perfused with precooled phosphate‐buffered saline (PBS) and then 4% paraformaldehyde (PFA; pH 7.4) for initial fixation. Brains were fixed overnight in 4% PFA at 4°C, and cryoprotected using successively 10%, 20%, and then 30% sucrose in PBS for 3 days. Ten‐micrometer‐thick coronal cryosections were obtained from each of three bregma levels (−1.5, 0, and +1.5 mm). Three brain sections per rat (with one brain section per bregma level) were affixed on Superfrost Plus slides (Menzel‐Glaser, Braunschweig, LS, Germany) and air dried overnight. The slides were boiled in citrate buffer (50 mM; pH 6.5) for 10 min to achieve antigen retrieval. After cooling, nonspecific binding was blocked with 10% goat serum at room temperature for 1 h. Brain sections were then incubated with the primary antibody in 3% goat serum at 4°C overnight, and then incubated with the corresponding secondary antibody at room temperature for detection of iNOS, cluster of differentiation (CD)‐68, and NeuN.

CD‐68 is a marker of macrophage/microglia (Chistiakov et al., 2017). As a neuronal nuclear antigen, NeuN labels surviving neurons. Slides were coverslipped with mounting medium 4′,6‐diamidino‐2‐phenylindole dihydrochloride (Invitrogen, Waltham, MA, USA). Five photomicrographs per bregma level were randomly taken over the right hemispheric areas at 100× magnification using a fluorescence microscope (Nikon, Tokyo, Japan). Number of positively stained cells was counted using Image J software (Image J, v1.51m9). Cell density was derived from the average number of positively stained cells per high‐power field, expressed as a percentage of that of the vehicle group and used for data analysis.

Primary antibody against CD‐68 (1:500) was purchased from AbD Serotec (Oxford, United Kingdom). Primary antibody against iNOS (1:100, sc‐651) was purchased from Santa Cruz Biotechnology. Primary antibody against NeuN (1:500, MAB377) was purchased from Merck Millipore (Massachusetts, USA). Alexa Fluor^®^488 goat anti‐mouse (1:500) and Alexa Fluor^®^594 mouse anti‐rabbit (1:500) were purchased from Merck Millipore.

### 2.11 Terminal deoxynucleotidyl transferase dUTP nick end labeling staining

The in situ cell death detection kit (Roche, Branchburg, NJ, USA) was used to detect TUNEL+ apoptotic cells. Brain sections obtained at the bregma levels were affixed on Superfrost Plus slides (Menzel‐Glaser) and air dried overnight. The slides were boiled in citrate buffer (50 mM; pH 6.5) for 10 min to achieve antigen retrieval. After cooling, 3% H_2_O_2_ was added as the blocking solution for 10 min before incubating with the freshly prepared permeabilization solution (0.1% Triton X‐100 in 0.1% sodium citrate) for 2 min on ice. Next, the terminal deoxynucleotidyl transferase dUTP nick end labeling (TUNEL) reaction mixture was prepared and applied to slides for 60 min. Five photomicrographs were randomly taken per brain section over the right hemispheric areas at 400× magnification using a fluorescence microscope (Nikon). Number of positively stained cells was counted using Image J software (Image J, v1.51m9). Cell density was derived from the average number of positively stained cells per high‐power field, expressed as a percentage of that of the vehicle group and used for data analysis.

### 2.12 Statistical analysis

All results were expressed in mean ± standard error of the mean (SEM). Number of rats used in each experiment was indicated in the text or figure legends, as appropriate. Student's *t*‐test was used to compare the differences between two groups. Analysis of variance (ANOVA) with Student–Newman–Keuls (SNK) as the post hoc test was used to compare the differences among three or more groups. Data on regional CBF, body weight changes, and rotarod latency over different time points were analyzed using two‐way repeated‐measures ANOVA followed by SNK, Tukey's honestly significant difference (HSD), or Games–Howell post hoc tests. SPSS 27 was used for all statistical analyses. A two‐tailed *p* < .05 was used to infer statistical significance.

## 3 RESULTS

### 3.1 T1DM aggravates infarct volume and neurological deficit after CIRI

**FIGURE 1:**
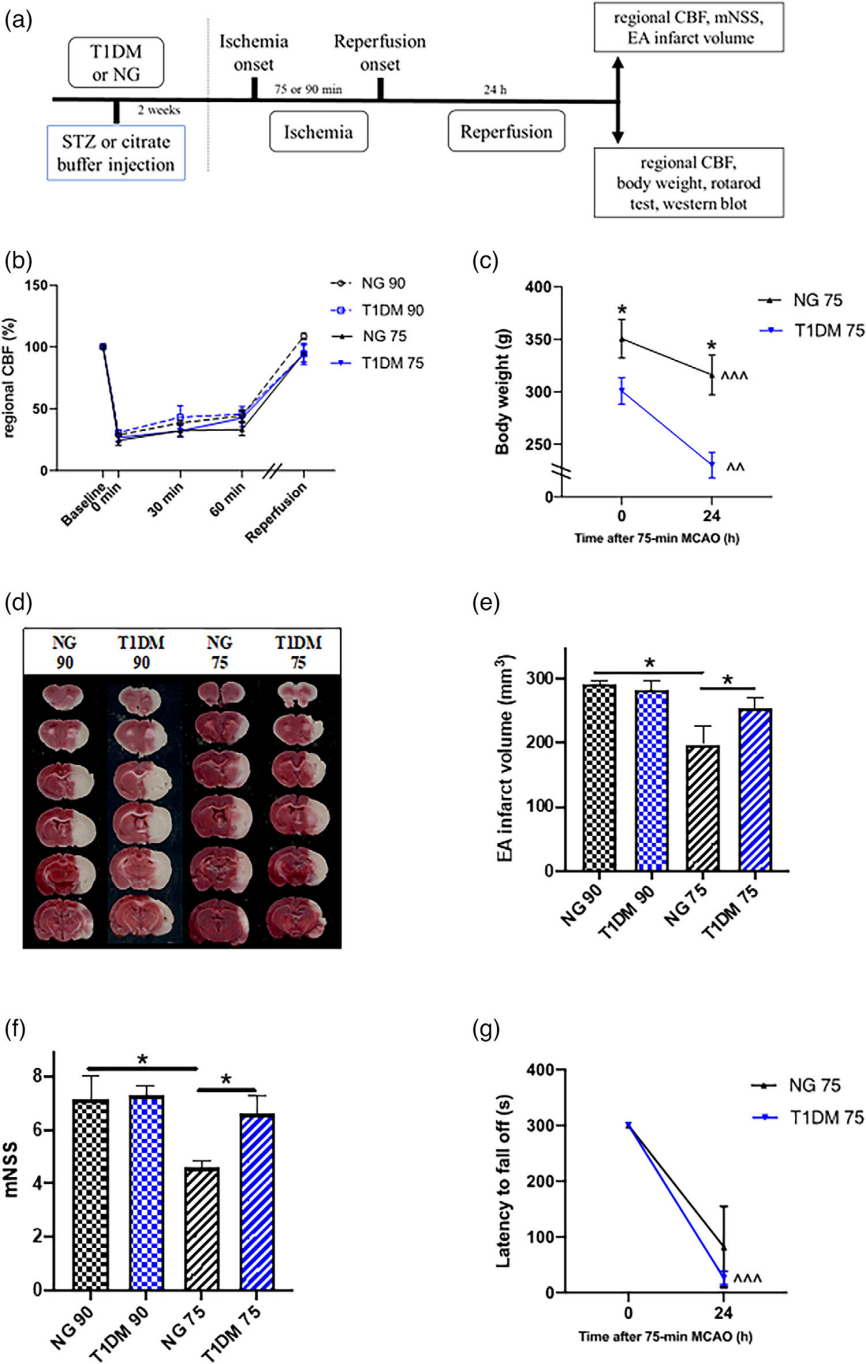
Effects of type 1 diabetes mellitus (T1DM) on regional cerebral blood flow (CBF), weight loss, infarct volume, and neurological deficit at 24 hours of cerebral ischemia–reperfusion injury (CIRI). (a) Experimental design with timeline. (b) Normalized regional CBF at different time points of 90‐ or 75‐min middle cerebral artery occlusion (MCAO) in normoglycemic (NG) (*n* = 6 in NG 90 and *n* = 6 in NG 75) and T1DM rats (*n* = 6 in T1DM 90 and *n* = 7 in T1DM 75); similar changes in regional CBF among all groups with reduction to less than 60% of the baseline level during ischemia (*p* < .01) and return to more than 80% of the baseline level upon reperfusion; data were analyzed by two‐way repeated‐measures ANOVA followed by Tukey's honestly significant difference (HSD) or Games–Howell post hoc test. (c) Body weight at baseline and 24 h of CIRI (*n* = 4 per group); data were analyzed by two‐way repeated‐measures ANOVA. (d) Representative photographs of coronal brain slices between bregma level +4 and −8 mm. (e) Edema‐adjusted (EA) infarct volume (*n* = 6 in NG 90, *n* = 6 in T1DM 90, *n* = 5 in NG 75, and *n* = 5 in T1DM 75); data were analyzed by one‐way ANOVA followed by SNK post hoc test. (f) Modified neurological severity score (mNSS) (*n* = 6 in NG 90, *n* = 6 in T1DM 90, *n* = 5 in NG 75, and *n* = 5 in T1DM 75); data were analyzed by one‐way ANOVA followed by Student–Newman–Keuls (SNK) post hoc test. (g) Latency to fall off in rotarod test at baseline and 24 h of CIRI (*n* = 4 per group); data were analyzed by two‐way repeated‐measures ANOVA. *p < .05 between groups; ^*p* < .05, ^^*p* < .01, and ^^^*p* < .001 between different time points of the same group.

STZ‐injected T1DM rats had higher blood glucose (31.9 ± 0.5 mmol/L, *n* = 35; *p* < .01) but lower body weight (313.7 ± 4.4 g, *n* = 30; *p* < .01) when compared to citrate buffer‐injected NG rats (8.7 ± 0.6 mmol/L, *n* = 10; 375.6 ± 16.6 g, *n* = 8). Experimental design of the study with timeline on CIRI in NG or T1DM rats is shown in Figure 1a. Changes in regional CBF were similar among all groups (with no significant intergroup difference); regional CBF decreased to less than 60% of the baseline level during ischemia (*p* < .01) and returned to more than 80% of the baseline level upon reperfusion (with no significant difference from baseline; Figure 1b). After 90‐min MCAO, death before sacrifice occurred more frequently in T1DM rats (45.5%; *n* = 11) than NG rats (14.3%; *n* = 7) despite similar EA infarct volume and mNSS in the surviving rats at 24 h post‐CIRI (Figure 1d–f). The mortality rate in T1DM rats before sacrifice decreased to 10.0% (*n* = 10) when the ischemic duration was reduced from 90 to 75 min. Therefore 75‐min ischemia followed by 24‐h reperfusion was used in the remaining study. NG rats had a smaller EA infarct volume and a lower mNSS at 24 h after 75‐min ischemia when compared to 90‐min ischemia (Figure 1d–f; *p* < .05). Post‐CIRI weight loss occurred in both NG and T1DM rats (*p* < .01 between baseline and 24 h post‐CIRI), and body weight of T1DM rats was significantly lower than that of NG rats at both baseline and 24 h of CIRI (Figure 1c; *p* < .05). T1DM resulted in a larger EA infarct volume (*p* < .05) and a higher mNSS (*p* < .05) when compared to NG rats at 24 h of CIRI (Figure 1d–f). Although latency to fall off was similarly reduced in both NG and T1DM rats at 24 h of CIRI, the reduction was significant in T1DM (Figure 1g; *p* < .001) but not NG rats; there was no difference between NG and TIDM rats at 24 h post‐CIRI.

### 3.2 T1DM enhances NF‐κB activation and cytochrome C release after CIRI

**FIGURE 2:**
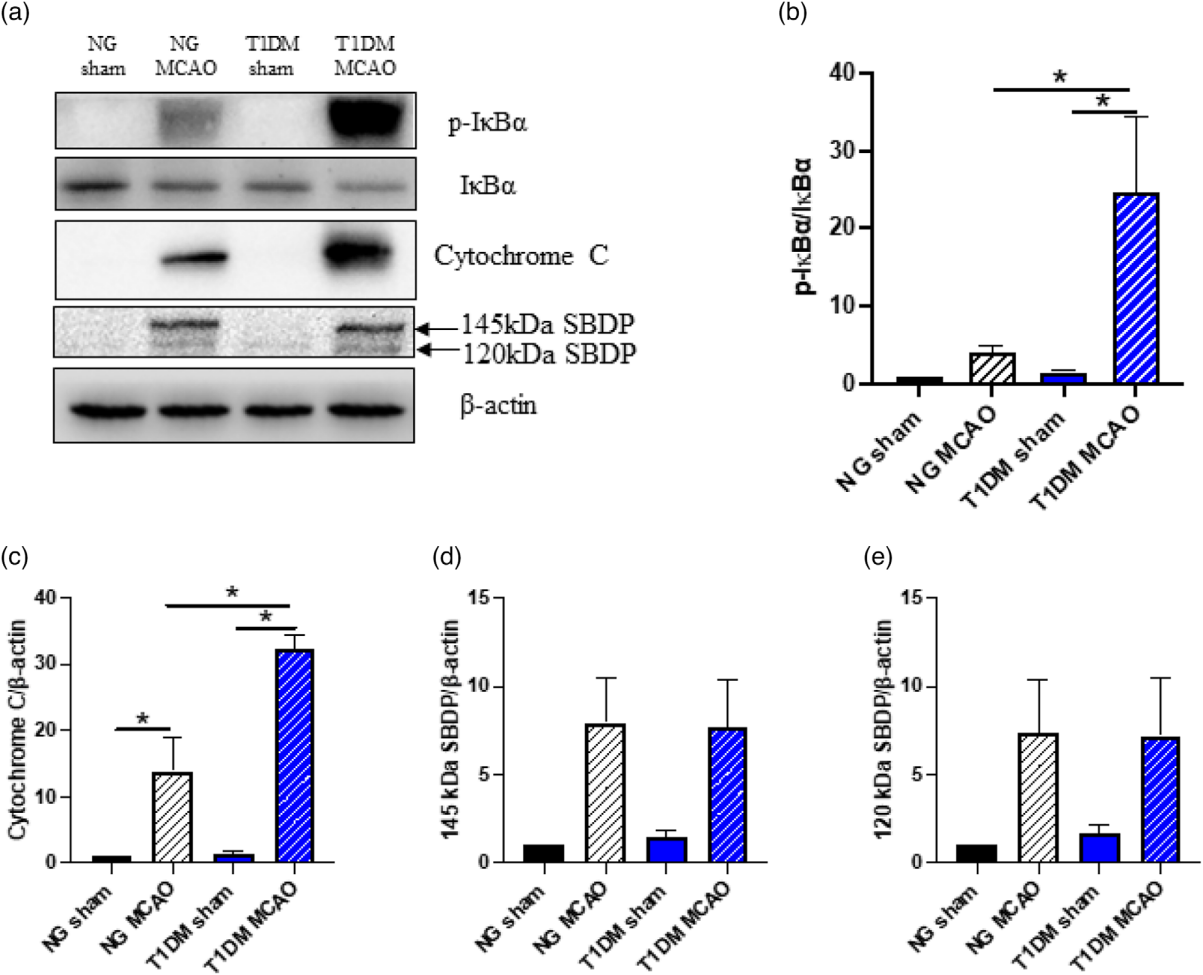
Effects of type 1 diabetes mellitus (T1DM) on protein expression in nuclear factor‐κB (NF‐κB) activation and apoptotic pathway at 24 h of cerebral ischemia–reperfusion injury (CIRI) achieved by 75‐min middle cerebral artery occlusion (MCAO) (*n* = 4 per group). (a) Representative western blot photographs of p‐IκBα, total IκBα, cytochrome C, 145‐kDa spectrin breakdown product (SBDP), 120‐kDa SBDP, and β‐actin. (b) p‐IκBα/total IκBα ratio derived from relative intensities of western blot bands. (c) Relative intensities of western blot bands for cytochrome C. (d) Relative intensities of western blot bands for 145‐kDa SBDP. (e) Relative intensities of western blot bands for 120‐kDa SBDP. Data were analyzed by one‐way ANOVA, followed by Student–Newman–Keuls (SNK) post hoc test as post hoc test. *p < .05 between groups. IκBα, NF‐κB inhibitor alpha; p‐IκBα, phosphorylated IκBα.

A representative western blot photograph of p‐IκBα, total IκBα, cytochrome C, 145‐kDa SBDP, 120‐kDa SBDP, and β‐actin is shown in Figure 2a. When compared to the sham group, CIRI from 75‐min ischemia plus 24‐h reperfusion led to an insignificant increase in p‐IκBα/total‐IκBα ratio (Figure 2b) and a significant increase in cytochrome C expression (Figure 2c; *p* < .05) in NG rats. T1DM per se did not affect the p‐IκBα/total‐IκBα ratio and cytochrome C expression at 24 h after sham CIRI. T1DM enhanced such elevations after CIRI so that both increased p‐IκBα/total‐IκBα ratio (Figure 2b; *p* < .05) and cytochrome C expression (Figure 2c; *p* < .05) were significantly different from the sham group as well as greater than those of NG rats. CIRI tended to increase the intensity of both 145‐ and 120‐kDa SBDP bands, the respective markers for activation of calpain and caspase‐3, at 24‐h reperfusion in both NG and T1DM rats (Figure 2d,e). ANOVA revealed a significant intergroup variation in 145‐kDa (*p* < .05) but not 120‐kDa SBDP band intensities. Nevertheless, there was no significant difference between the respective MCAO and sham groups under either NG or T1DM condition.

### 3.3 Melatonin protects against T1DM‐aggravated post‐CIRI worsening in infarct volume and neurological deficit

**FIGURE 3:**
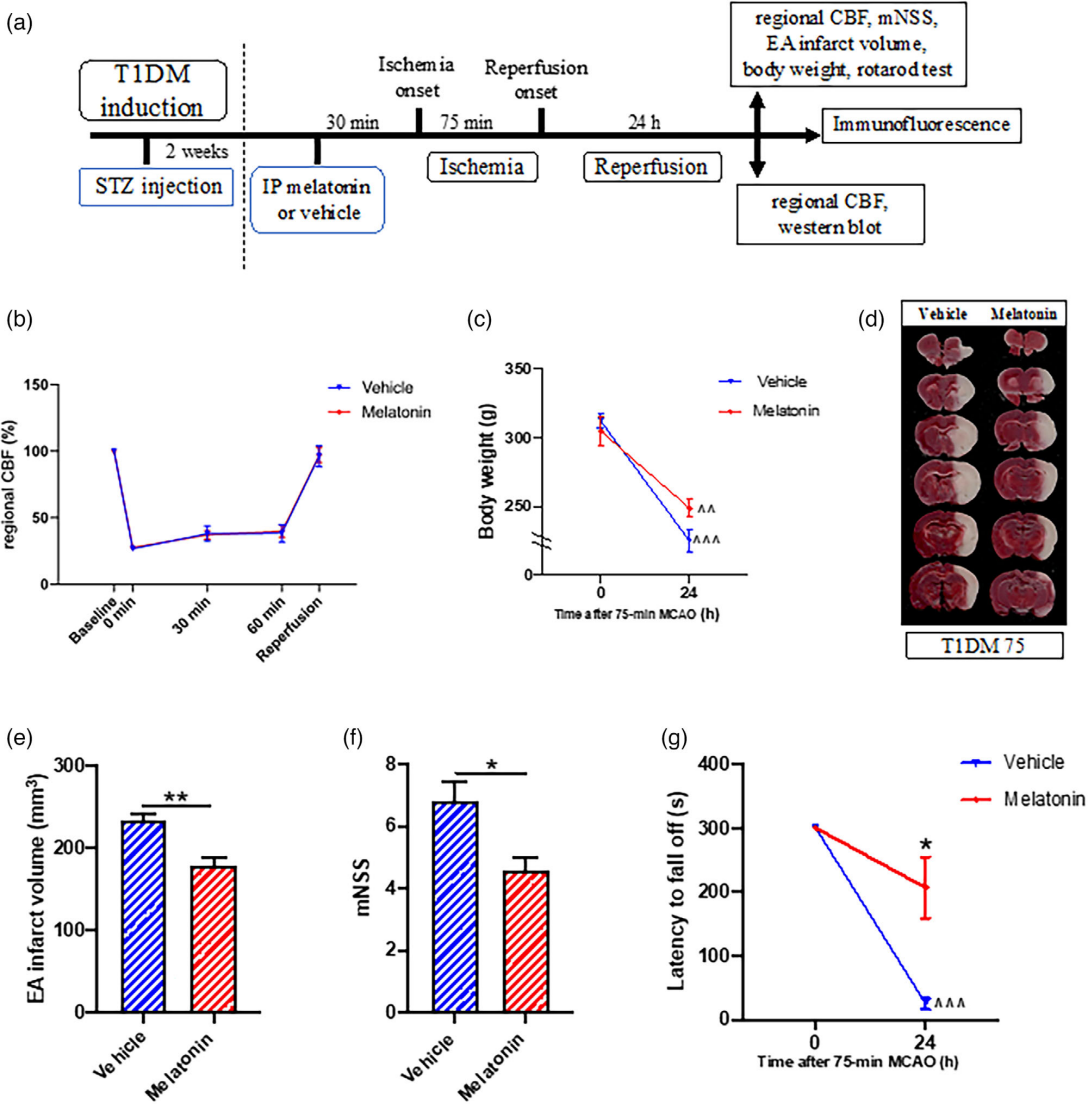
Effects of a single injection of melatonin (10 mg/kg given at 30 min before ischemia) on type 1 diabetes mellitus (T1DM)‐aggravated post‐cerebral ischemia–reperfusion injury (CIRI) changes in regional cerebral blood flow (CBF), weight loss, infarct volume, and neurological deficit at 24 h. (a) Experimental design with timeline. (b) Normalized regional CBF at different time points in vehicle (*n* = 7) and melatonin (*n* = 12) groups; data were analyzed by two‐way repeated‐measures ANOVA followed by Tukey's honestly significant difference (HSD) or Games–Howell post hoc test. (c) Body weight at baseline and 24 h of CIRI (*n* = 5 per group); data were analyzed by two‐way repeated‐measures ANOVA. (d) Representative photographs of coronal brain slices between bregma level +4 and −8 mm. (e) Edema‐adjusted (EA) infarct volume in vehicle (*n* = 6) and melatonin (*n* = 5) groups; data were analyzed by Student's *t*‐test. (f) Modified neurological severity score (mNSS) in vehicle (*n* = 6) and melatonin (*n* = 5) groups; data were analyzed by Student's *t*‐test. (g) Latency to fall off in rotarod test (*n* = 5 per group); data were analyzed by two‐way repeated‐measures ANOVA. *p < .05 and **p < .01 between groups; ^*p* < .05, ^^*p* < .01, and ^^^*p* < .001 between different time points of the same group.

Experimental design of the study with timeline on melatonin's protection against CIRI in T1DM rats is shown in Figure 3a. A single IP injection of melatonin (10 mg/kg) given at 30 min before ischemia onset did not affect changes in regional CBF during 75‐min MCAO (Figure 3b) or weight loss at 24‐h reperfusion (Figure 3c) but reduced the EA infarct volume (Figure 3d,e; *p* < .01) and mNSS (Figure 3f; *p* < .05) as well as lessened the reduction in latency to fall off in the rotarod test (Figure 3g; *p* < .05) at 24‐h reperfusion. Importantly, there was no significant reduction in latency to fall off in the melatonin‐treated rats at 24 h of CIRI. Melatonin treatment did not affect the blood glucose levels at 24‐h reperfusion (15.0 ± 2.6 mmol/L in vehicle‐treated rats and 15.1 ± 2.1 mmol/L in melatonin‐treated rats).

### 3.4 Melatonin exerts protective effects against T1DM‐aggravated post‐CIRI inflammation and apoptosis

**FIGURE 4:**
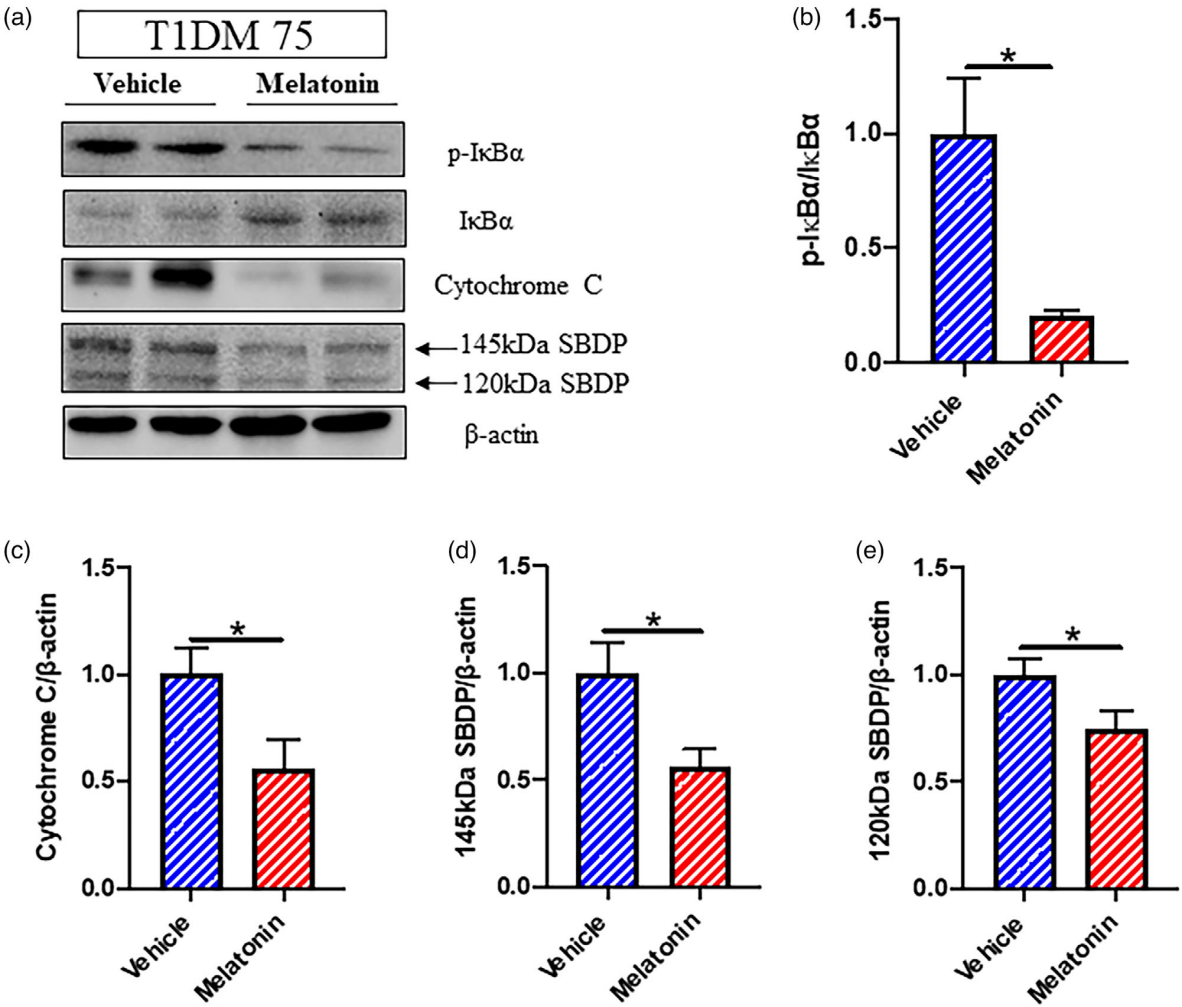
Effects of a single dose of melatonin (10 mg/kg given at 30 min before ischemia) on type 1 diabetes mellitus (T1DM)‐aggravated protein expression in nuclear factor‐κB (NF‐κB) activation and apoptotic pathways at 24 h of cerebral ischemia–reperfusion injury (CIRI) achieved by 75‐min middle cerebral artery occlusion (MCAO). (a) Representative western blot photographs of p‐IκBα, total IκBα, cytochrome C, 145‐kDa spectrin breakdown product (SBDP), 120‐kDa SBDP, and β‐actin. (b) p‐IκBα/total IκBα ratio derived from relative intensities of western blot bands (*n* = 4 per group). (c) Relative intensities of western blot bands for cytochrome C (*n* = 8 per group). (d) Relative intensities of western blot bands for 145‐kDa SBDP (*n* = 8 per group). (e) Relative intensities of western blot bands for 120‐kDa SBDP (*n* = 8 per group). *p < .05 between groups using Student's *t*‐test. IκBα, NF‐κB inhibitor alpha; p‐IκBα, phosphorylated IκBα.

**FIGURE 5:**
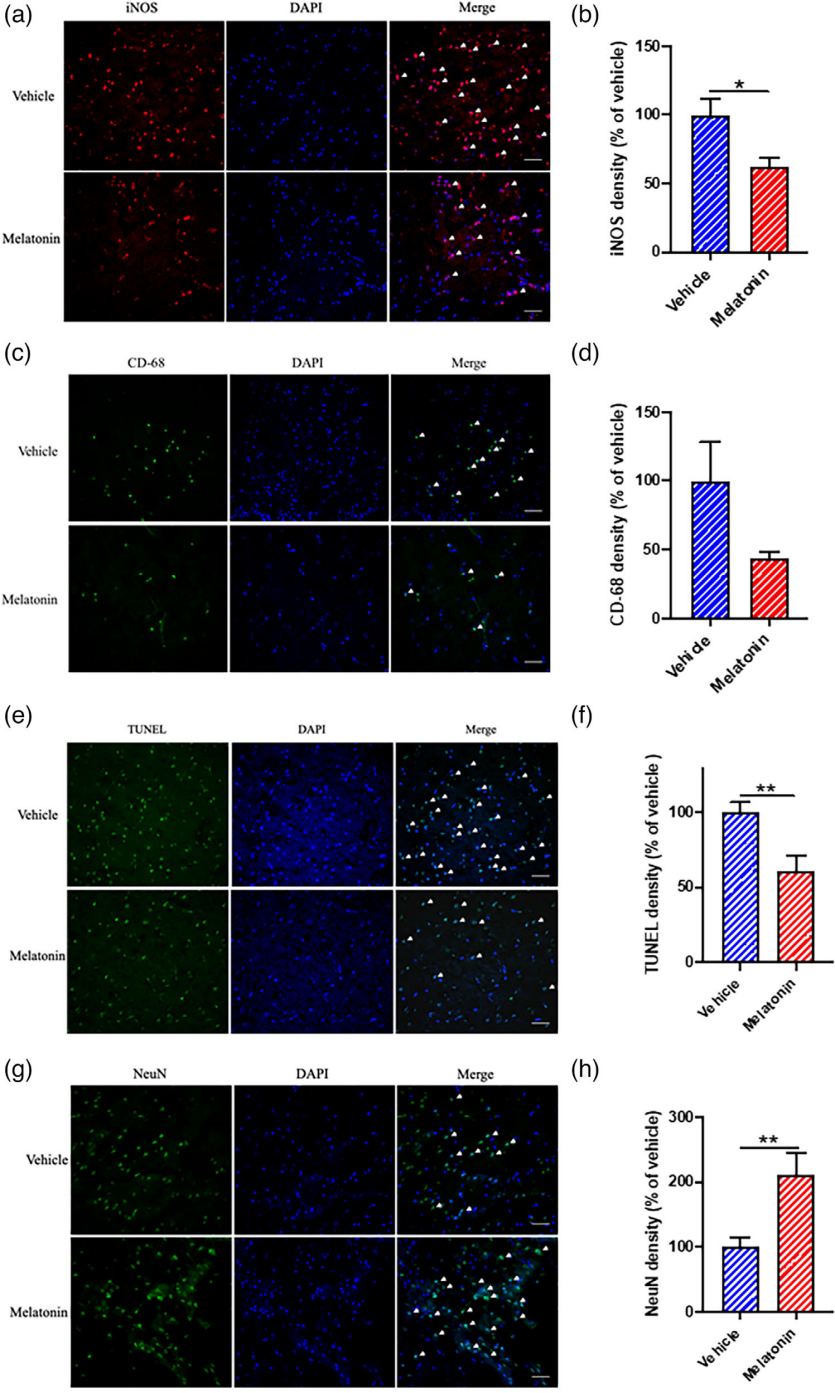
Effects of a single dose of melatonin (10 mg/kg given at 30 min before ischemia) on type 1 diabetes mellitus (T1DM)‐aggravated inflammatory and apoptosis markers using immunofluorescence staining at 24 h of cerebral ischemia–reperfusion injury (CIRI) achieved by 75‐min middle cerebral artery occlusion (MCAO). (a) Representative fluorescence photomicrographs of iNOS+ cells (scale bar = 50 μm). (b) Relative iNOS+ cell density in vehicle (*n* = 6) and melatonin (*n* = 5) groups. (c) Representative fluorescence photomicrographs of cluster of differentiation (CD)‐68+ cells (scale bar = 100 μm). (d) Relative CD‐68+ cell density in vehicle (*n* = 6) and melatonin (*n* = 5) groups. (e) Representative fluorescence photomicrographs of TUNEL+ apoptotic cells (scale bar = 50 μm). (f) Relative TUNEL+ cell density in vehicle (*n* = 4) and melatonin (*n* = 5) groups. (g) Representative fluorescence photomicrographs of neuN+ surviving neurons (scale bar = 50 μm). (h) Relative NeuN+ cell density in vehicle (*n* = 6) and melatonin (*n* = 5) groups. *p < .05 and **p < .01 between groups using Student's *t*‐test.

A representative western blot photograph of p‐IκBα, total IκBα, cytochrome C, 145‐kDa SBDP, 120‐kDa SBDP, and β‐actin is shown in Figure 4a. When compared to vehicle treatment in T1DM rats, a single IP injection of melatonin (10 mg/kg) given at 30 min before ischemia onset led to a lower p‐IκBα/total‐IκBα ratio (Figure 4b; *p* < .05), a reduced cytochrome C release (Figure 4c; *p* < .05), decreased 145‐kDa SBDP band intensity (Figure 4d; *p* < .05), and decreased 120‐kDa SBDP band intensity (Figure 4e; *p* < .05) at 24 h of CIRI as evaluated using western blot. In addition, the treatment led to decreased density of iNOS+ cells (Figure 5a,b; *p* < .05) and tended to reduce density of CD‐68+ cells (Figure 5c,d) in T1DM rats at 24 h of CIRI using immunofluorescence staining. Furthermore, the treatment led to a reduced density of TUNEL+ apoptotic cells (Figure 5e,f; *p* < .01) and an increased density of NeuN+ neuronal cells (Figure 5g,h; *p* < .01) at 24 h of CIRI.

## 4 DISCUSSION

Most experimental studies on CIRI use healthy animals without vascular risk factors (Dirnagl et al., 1999; Fluri et al., 2015), and DM is a common and important risk factor (Campbell et al., 2019). Random blood glucose is higher, and body weight is lower in STZ‐injected rats than in citrate buffer‐injected rats, indicating successful induction of T1DM. An ischemic duration of 90 min is commonly used in MCAO studies (Liu et al., 2009). Premature mortality before reaching 24‐h reperfusion time point is much higher in T1DM rats than in NG rats, while the surviving T1DM rats have neither a larger infarct nor worse neurological deficit when compared to NG rats at 24‐h reperfusion. It is plausible that rats with a larger infarct plus worse cerebral edema would die prematurely. A shorter ischemic duration of 75 min has led to a reduced premature mortality rate in T1DM rats with infarct size and neurological deficit similar to those of NG and T1DM rats after 90‐min ischemia. Nevertheless, 75‐min ischemia in NG rats has resulted in a smaller infarct and milder neurological deficit when compared to NG rats after 90‐min ischemia. Our findings in NG rats are in line with the published observations that CIRI severity is dependent on ischemic rather than reperfusion duration (Li et al., 2013). After 75‐min ischemia and 24‐h reperfusion, a larger brain infarct volume and worse neurological deficit are seen in T1DM rats when compared to NG rats. These findings are in line with the published observations that T1DM aggravates CIRI severity (Jouihan et al., 2013; Toung et al., 2000; Ye et al., 2011). Weight loss has occurred after CIRI in both NG and T1DM rats so that T1DM rats are lighter than NG rats at both baseline and 24 h after 75‐min ischemia. Studies have shown a positive correlation between infarct volume and weight loss after acute stroke (Cai et al., 2015). A greater weight loss in T1DM rats is probably related to the increased severity of CIRI when compared to NG rats. Latency to fall off in rotarod test is similarly shortened at 24 h post‐75‐min ischemia in both NG and T1DM rats, but the reduction is significant in T1DM rats only because of a wide variation in the results of NG rats. When compared with mNSS, rotarod test is more sensitive to mild impairment in balance, coordination, and motor planning (Hamm et al., 1994; Rustay et al., 2003).

Stroke patients with admission hyperglycemia have poor clinical outcome. While previous experimental studies have reported increased brain infarct volume in T1DM rats after CIRI (Jouihan et al., 2013; Toung et al., 2000; Ye et al., 2011), the mechanisms underlying the detrimental effects of hyperglycemia on CIRI remain elusive. The oxidizing environment with elevated tissue carbohydrate concentration under DM condition aggravates the formation of AGEs via hyperglycemia‐driven nonenzymatic glycation modifications of proteins, lipids, or nucleic (Goldin et al., 2006; Vincent et al., 2011). AGEs lead to several microvascular and macrovascular complications through cross‐linking of molecules within the basement membrane of the extracellular matrix and via stimulation of the RAGE (Goldin et al., 2006). AGE–RAGE signaling activates transcription factor NF‐κB and upregulates its target genes. Besides, AGEs block endothelial NOS activity and contribute to ROS generation (Goldin et al., 2006). Furthermore, glucose serves as the electron donor during reperfusion‐induced superoxide generation in neuronal cultures (Bemeur et al., 2007). Oxidative stress, an outcome of excessive generation of ROS and repression of antioxidants, also contributes to the pathogenesis of DM and its complications, creating a vicious cycle (Kang & Yang, 2020). Excessive accumulation of ROS in the presence of suppressed antioxidant system induces mitochondrial damage, cellular apoptosis, and inflammation (Kang & Yang, 2020). The NF‐κB pathway is activated during ischemia/reperfusion injury (Du et al., 2019). The resulting pro‐inflammatory cytokines can further stimulate NF‐κB to generate other pro‐inflammatory substances (cytokines, chemokines, and adhesion molecules) (Lawrence, 2009). Significantly increased expression of p‐IκBα/IκBα indicates post‐CIRI activation of NF‐κB pathway in T1DM but not NG rats. T1DM per se would not lead to NF‐κB pathway activation after sham MCAO, revealing the importance of specific triggers such as CIRI.

In the intrinsic apoptotic pathway, cerebral ischemia elevates cytosolic calcium levels, which will activate calpains and mediate cleavage of Bcl‐2 interacting domain (BID) to truncated BID (tBID). tBID interacts with apoptotic proteins such as Bad and Bax at the mitochondrial membrane, which is neutralized by anti‐apoptotic Bcl‐2 family proteins Bcl‐2 or Bcl‐XL (Broughton et al., 2009). Bcl‐2 functions as anti‐apoptosis via preventing the efflux of cytochrome C from mitochondria (Yang et al., 1997). After heterodimerization of pro‐apoptotic proteins with tBID, mitochondrial transition pores are opened, leading to release of cytochrome C as an apoptosis‐inducing factor. Cytosolic cytochrome C binds with apoptotic protein‐activating factor‐1 and pro‐caspase‐9 to form an apoptosome, which will then activate caspase‐3, a key mediator of apoptosis in ischemic stroke (Broughton et al., 2009). When compared to NG rats undergoing sham MCAO, there is increased expression of cytochrome C in NG rats after CIRI, T1DM per se has no effect, and this post‐CIRI increase is aggravated by T1DM condition. Apart from its central role in apoptosis, mitochondrial release of cytochrome C blocks electron transport, leading to reduced adenosine triphosphate (ATP) generation, ATP depletion, and, finally, necrosis (Kang, 2001). Previous studies have reported increased activation of NF‐κB with increased production of proinflammatory cytokines in T1DM condition and suppression of prediabetic cytokine production by a NF‐κB inhibitor (Shao et al., 2015; Triñanes et al., 2012). Other studies have shown increased apoptosis in DM condition (Ho et al., 2013). Nevertheless, there is little information on the role of NF‐κB activation and pro‐apoptosis in hyperglycemia‐aggravated CIRI. The present study has provided some novel findings on the participation of NF‐κB activation and increased cytochrome C release in aggravation of CIRI under T1DM condition. CIRI tends to increase calpain‐mediated SBDP and caspase‐3‐mediated SBDP, and these insignificant post‐CIRI changes are not affected by T1DM condition. Thus, our results do not support an important role of calpain activation and caspase‐dependent apoptosis in aggravation of CIRI under T1DM condition.

As a neurohormone, melatonin plays a role in energy homeostasis, sleep–wake cycle, and biological rhythms (Amaral et al., 2014). It possesses neuroprotective effects in various neurological diseases, including Parkinson's disease, multiple sclerosis, Alzheimer's disease, and stroke (Alghamdi, 2018). Melatonin, given either before or after ischemia, alleviates CIRI, leading to smaller infarct volume, decreased brain edema, better neurological scores, increased neuronal survival, and enhanced neurogenesis (Feng et al., 2017; Kilic et al., 2008; Yang et al., 2015). In previous studies using nondiabetic rats, a single injection of melatonin at doses of 1.5, 5, 15, or 50 mg/kg has been used to evaluate its effectiveness against ischemic stroke. Melatonin dose between 5 and 15 mg/kg protects against focal cerebral ischemia (Pei et al., 2002). Evaluation of melatonin at doses of 20 or 50 mg/kg in a rat model of intracerebral hemorrhage has failed to find any beneficial effect on hematoma size and apoptosis at 72 h (Leung & Cheung, 2021). In the planning of the present study, melatonin doses of 5 and 10 mg/kg were used. Melatonin at 10 mg/kg was more effective than 5 mg/kg with a respective reduction of EA brain infarct volume by 23.1% and 15.9% (data not shown). Thus, melatonin at 10 mg/kg was chosen for this study.

The underlying beneficial mechanisms include reduced NF‐κB expression (Liu et al., 2017; Zhao et al., 2018) and decreased apoptosis (Fernández et al., 2015; Naveenkumar et al., 2020; Zhai et al., 2017). Melatonin may also have indirect relationship with DM. A lower level of melatonin is associated with an increased risk of DM (Mcmullan et al., 2013). Melatonin supplementation may be beneficial in hyperglycemic conditions (Amaral et al., 2014). However, there are little data on its effects against CIRI in diabetic animals.

Our current results have provided for the first time some evidence on neuroprotective effects of melatonin on CIRI in T1DM rats. A single IP dose of melatonin at 10 mg/kg given 30 min before ischemia onset decreases the EA brain infarct volume, neurological deficits, and body weight loss and improves rotarod performance when compared with the vehicle group without influencing blood glucose level. The beneficial mechanisms of melatonin treatment on CIRI in hyperglycemic conditions include reduction in inflammation, apoptosis, and neuronal cell death. Reduced inflammation is reflected by a reduction in density of iNOS+ cells and an insignificant reduction in infiltration of CD‐68+ macrophage/microglia. Decreased TUNEL+ cell density indicates a reduction in apoptotic process. A higher density of NeuN+ cells reflects a decreased neuronal cell death. As T1DM aggravates CIRI partly via further activation of NF‐κB pathway activation and enhanced mitochondrial cytochrome C release, decreased expressions of p‐IκBα/IκBα and cytochrome C in melatonin‐treated rats when compared to vehicle‐treated rats may represent important beneficial effects of melatonin. Although calpain activation and caspase‐dependent apoptosis may not play an important role in aggravation of CIRI under T1DM condition, melatonin administration can suppress calpain activation and caspase‐dependent apoptosis. Further studies are warranted.

Melatonin confers many neuroprotective effects via both receptor‐mediated and receptor‐independent mechanisms (Genade et al., 2008; Hardeland, 2018; Liu et al., 2016; Reiter et al., 2016; Tamtaji et al., 2019). Selective MT1 receptor agonists are potential therapeutic agents in Huntington's disease (Liu et al., 2016). MT2 activation can enhance neurogenesis in mice with CIRI (Liu et al., 2016). A higher density of NeuN+ cells after melatonin treatment was seen in the present study. As a potent and high‐capacity free radical scavenger, melatonin possesses receptor‐independent beneficial effects such as an inhibition in cataract formation, a decrease in oxidative stress from hyperbaric hyperoxia, an amelioration in hyperthyroidism, and a reduction in the toxicity of sepsis and septic shock (Reiter et al., 2007). A reduction in density of iNOS+ cells and CD‐68+ macrophage/microglia after melatonin treatment was seen in the present study. To further elucidate the underlying protective mechanisms of melatonin in T1DM rats with CIRI, selective melatonin receptor blockers such as luzindole (Genade et al., 2008) and genetic deletion of the MT1 and/or MT2 receptors can be used in future studies using appropriate in vitro or in vivo models of CIRI (Liang et al., 2021; Liu et al., 2016).

In conclusion, T1DM aggravates CIRI partly via further activation of NF‐κB pathway activation and enhanced mitochondrial cytochrome C release. A single IP dose of melatonin at 10 mg/kg given 30 min before ischemia onset is neuroprotective against CIRI in T1DM rats. The beneficial mechanisms of melatonin treatment include reduction in inflammation, apoptosis, and neuronal cell death especially via stabilization of NF‐κB pathway and mitochondrial cytochrome C release.

## AUTHOR CONTRIBUTIONS

Qian Xu and Raymond Tak Fai Cheung were involved in development of the hypotheses and experimental design. Qian Xu conducted the majority of the experiments. Qian Xu and Raymond Tak Fai Cheung performed the data analyses. Qian Xu drafted the manuscript. Raymond Tak Fai Cheung critically revised the manuscript. Qian Xu and Raymond Tak Fai Cheung approved the final version of the manuscript.

## CONFLICT OF INTEREST STATEMENT

The authors declare no conflicts of interest.

### PEER REVIEW

The peer review history for this article is available at https://publons.com/publon/10.1002/brb3.3118

## Data Availability

The data that support the findings of this study are available on request from the corresponding author. The data are not publicly available due to privacy or ethical restrictions.
